# Can Medical Decision-making at the Scene by EMS Staff Reduce the Number of Unnecessary Ambulance Transportations, but Still Be Safe?

**DOI:** 10.1371/currents.dis.f426e7108516af698c8debf18810aa0a

**Published:** 2015-06-30

**Authors:** Mahmoudreza Peyravi, Per Örtenwall, Amir Khorram-Manesh

**Affiliations:** Prehospital and Disaster Medicine Centre, Sahlgrenska Academy, Gothenburg University, Gothenburg, Sweden; Department of Medical Informatic Management, Shiraz University of Medical Sciences, Shiraz, Iran; Pre-hospital and Disaster Medicine Centre, Department of Surgery, Institute of clinical sciences, Sahlgrenska Academy, Gothenburg University, Gothenburg, Sweden; Pre-hospital and Disaster Medicine Centre, Department of Surgery, Institute of clinical sciences, Sahlgrenska Academy, Gothenburg University, Gothenburg, Sweden

**Keywords:** Ambulance Transportation, decision-making, Discharging from the Scene, EMS, Outcome

## Abstract

Objectives: The aim of this study was to evaluate the procedures adopted by the staff of the Shiraz Emergency Medical Services (EMS) and the outcome of the patients discharged from the scene over a one-year period.

Background: Unnecessary use of ambulances results in the overloading of EMS and the over-crowding of emergency departments. Medical assessment at the scene by EMS staff may reduce these issues. In an earlier study in Shiraz, 36% of the patients were left at home/discharged directly from the scene with or without treatment by EMS staff after consulting a physician at the dispatch center. However, there has been no evaluation of this system with regard to mortality and morbidity.

Materials and Methods: Retrospective data on all missions performed by the Shiraz EMS (2012-2013) were reviewed. All the patients discharged from the scene by the EMS staff on the 5th, 15th, and 25th days of each month were included. A questionnaire with nine questions was designed, and available patients/relatives were interviewed prospectively (2014; follow-up period 4-12 months).

Results: Out of 3019 cases contacted, 994 (almost 33%) replied. There were 26%-93% reductions in the complaints in all disease categories. A group of the patients left the scene at their own will. Of those who were discharged by the EMS staff at the scene, over 60% were without any complaints. Twelve out of 253 patients died after they were sent home by the EMS staff.   Conclusions: Patients may be discharged at the scene by EMS staff and after consulting a physician. However, there is a need for a solid protocol to ensure total patient safety. This calls for a prospective study.

## Introduction

Emergency Medical Services (EMS) are the frontline services for patients’ care in peacetime and during major incidents 1. EMS availability is one of the major factors in achieving successful management of patients. Such availability is possible either by increasing the number of ambulances or reducing the number of unnecessary missions by screening the patients who need transportation, based on their disease severity. The unnecessary use of ambulances results in the overloading of EMS as well as the over-crowding of emergency departments (EDs) 2. In many countries, new strategies have been implemented in order to make the public aware of the proper use of EMS as well as EDs 3 ^,^ 4. One way to prevent these problems is to assess the patient at the scene and decide on the need for ambulance transportation to a selected facility. Standardized treatment plans have been implemented for specific diagnoses such as cardiac and hip-fractures to bypass EDs.

The most important tasks for EMS are the four Ts (i.e. Time, Triage, Treatment, and Transportation) 1 ^,^ 5 . Shorter response time may save lives 2 ^,^ 6 ^,^ 10 . Triage is initiated by a medical dispatch center in many countries and constitutes the first step to selecting the right patient for the right transportation to the right healthcare facility. Based on medical protocols, patients are triaged/prioritized to different levels of urgency, and each priority should be transported within a set time frame 10 . Earlier studies have shown a discrepancy between first triage made by the dispatch center and the one made by EMS crews at the scene 10 ^,^ 11 . The high number of over-triage in these studies indicated the over-utilization of ambulance transportation to hospitals, limiting ambulance availability and contributing to ED overcrowding. Therefore, a correct and validated diagnosis at the scene may confer a better use of resources 12 ^-^ 15. As a consequence, some patients will be denied ambulance transportation to the hospital, which is not authorized in some countries. It is also a huge challenge for EMS staff to make a decision at the scene since their ability and skills for making such decisions as compared to a general practitioner have been questioned 11 ^,^ 16 ^,^ 17 .

In Iran, EMS crews can opt against transportation to the hospital from the scene after assessing the patient. Their findings, after a primary examination, are discussed with a physician at the dispatch center, who decides whether the patient should be transported to the hospital or not, however, no special protocol is used. In a previous report made by this group, 36% of the patients were discharged directly from the scene with or without treatment by the Shiraz EMS staff, based on this procedure. Nevertheless, no evaluation of this system regarding mortality and morbidity has ever been conducted 17.

Since the outcome of such decision-making may have an impact on future guidelines in emergency care and ambulance activities 18 ^-^ 20, we aimed to evaluate the procedures adopted by the Shiraz EMS and the outcome of the patients discharged from the scene over a one-year period (i.e. 2012-2013).

## Materials and Methods

**Data collection**

All patients subject to EMS missions in Shiraz (n = 81999), over a one-year period (i.e. March 21, 2012 to March 20, 2013), were retrospectively identified and their data (age, gender, main complaint, primary diagnosis on the scene, etc.) were reviewed. A sample of all the patients discharged from the scene by the Shiraz EMS (n= 3019) was obtained from the 5th, 15th, and 25th days every month. These patients were contacted by telephone 4 to 12 months after they were discharged. If the patients were not available, their relatives/next of kin listed were contacted. Three attempts were made to get in touch by telephone. No other methods were used (e.g. letter). All the questionnaires were sorted by numbers, and the patients’ data were not available to the main author at the time of review. The patients who replied were asked to answer the following questions by free text.

-What were the main reasons to request an ambulance and your chief complaint?

-What were your symptoms?

-Was the decision not to be transported to the hospital your own, or did the ambulance crew make it?

-If it was the staff’s decision, were you satisfied with it?

-Did your symptoms continue after being discharged? If your answer is yes, what were those?

-Did you visit a clinic or hospital ∕physician after being discharged by EMS?

-What was their diagnosis ∕opinion when visiting the clinic or hospital?

-What was the treatment?

-Have you recovered?

**Ethical permission**

This study was approved by the Ethics Committee of Shiraz Medical University (2011-100/7 Feb. 2011).

**Statistics**

Data analysis was conducted using Statistical Package for the Social Sciences (SPSS) version 20 (SPSS Inc., Chicago, Illinois, USA). The data analysis is expressed using descriptive statistics, including range and mean ± Standard Deviation (SD). The frequency and percentage of the categorical data are presented.

## Results

In total, 3019 patients who were directly discharged from the scene were identified. Of these, 994 (33%) patients could be followed up. No information could be obtained for the remaining 2025 patients due to:

1. 1469 patients (49 %) did not reply to our calls.

2. 90 patients (3 %) were unwilling to cooperate.

3. 91 patients (13 %) could not remember their incidents.

4. 85 missions were cancelled because patients changed their mind or were not available on the scene.

Information about major complaints leading to an ambulance call was available for 935 out of the 994 patients. Table 1 shows these complaints divided into eight categories and whether the complaint existed after the EMS management at the scene. As is shown in Table 1, there was a reduction in the number and rate of complaints after the EMS engagement (26% to 93%). The most impressive reduction concerned neurological and psychiatric cases, and the least reduction was seen among the patients with medical, toxic, and circulation complaints, followed by surgical and respiratory cases.

**Table 1. Major reasons/complaints for calling an ambulance:** 

Before ambulance arrival			After discharge from the scene		
Categories	Number	%	Number	Difference in number	% reduction
Trauma	67	7.2	24	43	64
Toxic	31	3.3	20	11	35
Neurology	212	22.7	14	198	93
Circulation	138	14.8	50	88	36
Medical	337	36	247	90	26
Respiratory	131	14	56	75	57
Surgery	13	1.4	6	7	54
Psychiatry	6	0.6	1	5	83

Information about decision-making at the scene and follow-up was available for 879 out of the 994 patients. In 71.2% (626 cases), the recipients made the decision not to be transferred to the hospital after the EMS staff’s arrival. In most cases, the symptoms had subsided or the patient did not want any help from the staff due to other reasons. In 28.8% (253 cases), the EMS staff discharged the patients from the scene of incidents or left them at their home after definitive treatment or advice. A primary diagnosis was made, and recommendations such as self-care guidance or later and planned visits to a healthcare center were given. In 7 cases, the patients were recommended to be transported to the hospital, but rejected, not favoring the hospital suggested by the EMS staff. Table 2 depicts the distribution of the patients after scene examination by the EMS staff.

The patients who rejected transportation by their own decision (626 out of 994, [63%]) filled in a release sheet before being discharged from the scene. Of the remaining 368 patients, 253 (25.5%) were discharged from the scene by the EMS and in 27 (2.7%) patients no information was available. Among the patients discharged by the EMS, 34 (13.5%) were recommended to seek healthcare privately due to the non-urgent nature of their complaints. Another 18 (7.1%) patients were transported to a clinic or hospital due to insufficient treatment at the scene. Around 43.9% of the patients (n = 436 out of 994) were male and 386 (38.8%) female. In the remaining 172 (17.3%) patients, no information about the gender could be found.

For 442 (44.5%) patients, the symptoms remained unchanged after they were discharged from the scene, while the symptoms subsided in 489 (49.2%). Of the remaining 6.3%, 4.3% (n = 43) did not reply to this question and 2% (20) were dead. As is shown in Table 2, a sum of 229 patients had still complaints after going home by their own decision compared to 75 out of the 253 patients discharged by the EMS (36.6% vs. 29.7%). On the other hand, 358 of those who decided to go home had no complaint in their follow-up versus 166 who were discharged by the EMS staff (57.2% vs. 65.6%). There was a statistically significant difference in favor of the decisions made by the EMS staff according to the chi-square test (P < 0.001).

**Table 2. Patients’ status at the time of investigation:** 

Decision-maker	Current situation	Number	Percent
Patient (n=626)	With Problem	229	36.6
	Without problem	358	57.2
	Dead	39	6.2
EMS (n=253)	With Problem	75	29.7
	Without problem	166	65.6
	Dead	12	4.7
			
Total (n=994)	With Problem	304	30.6
	Without problem	524	52.7
	Dead	51	5.1
	Unknown	115	11.6

In this study, 51 patients died after being discharged from the scene. Of these, 39 belonged to those who left the scene by their own decision compared to 12 out of the 253 patients who were discharged by the EMS staff at the scene. Although the number of deaths was lower in the latter group (discharged at the scene by the EMS), there was no statistically significant difference in mortality between these two groups (P < 0.5). The causes of deaths were not available since no autopsies are performed in Iran due to current practices, unless a crime is suspected.

## Discussion

The major tasks of EMS are short time response to the scene, accurate triage, proper treatment, and quick and safe transfer to the hospital 19 ^,^ 20. In an earlier study of the Shiraz EMS 17, we succeeded in determining that one third (36%) of the patients were not sent to the hospital. Iranian EMS staff has the authority to discharge a patient from the scene after consultation with a physician at the dispatch center^17^. In this study, we followed up a group of discharged patients, prospectively, 4-12 months after EMS missions. The low response rate (33%) despite numerous attempts to contact the patients illustrates the difficulties in conducting prehospital research and the follow-up of prehospital cases.

The results obtained in this study imply that a large number of patients requesting an ambulance are non-emergency cases and can be treated at places other than hospitals. This study also shows that by giving the right mandate and medical support to EMS staff, there is a possibility to treat and discharge a proportion of the patients directly from the scene. An overuse of ambulances leads to decreased availability of ambulances and ED overcrowding, thus limiting the possibility of offering emergency care to more severely ill patients 2 ^,^ 3 ^,^ 21. Although some studies criticize discharging patients at the scene by EMS staff due to patient safety issues, it is logical to assume that a better cooperation between EMS staff and a consultant physician and training and availability of a consultant physician may increase the accuracy and safety of such decision-making 22 ^,^ 23 . This strategy will be specifically useful in low-middle-income countries, where the infrastructure and human resources are inadequate 24 ^-^ 26. A better standard in decision-making, such as a guideline or a protocol, can make this strategy more fruitful 27 ^-^ 29.

In this study, there was a 5.1% mortality rate in total, but the rate of mortality was the same in both groups of patients, i.e. those who decided to go home by their own decision and those who were left at home/discharged by the EMS. Even if the EMS personnel were actively involved in the latter group, the impact of their presence or advice to the patients in the first group cannot be neglected. Whether this mortality rate is acceptable or not depends much on the real cause of the death, which was not available and thus neither comparison nor any conclusion can be made. However, it is a good assumption that increased educational levels of EMS or staffing ambulances with a physician together with an evidence-based protocol will increase the possibility of a better selection and correct diagnosis, as has been shown recently^30^.

In this study, there was a marked reduction in the number of complaints, especially in neurological and psychiatric cases, followed by trauma. One explanation for this finding is that maybe the majority of the calls were non-emergencies and minor injuries, which could be handled by the EMS staff.

**Conclusion**

We advocate a correct selection of patients discharged from the scene or left at home by EMS. Using a standardized protocol, which eliminates the bias made by different staff and physicians may safeguard this process 29. The results could be indicative for a prospective study and have an impact to improve the process and selection of the patients that should be transported to the hospital or can safely be discharged directly.

**Limitation**

One limitation of this study is the lack of a 30-day follow-up of the patients after being discharged at the scene and the lack of autopsy results. In both cases, the absence of registration and guidelines are evident. Moreover, the low number of the respondents (irrespective of the calls) during the follow-up renders the statistical analysis harder and the obtained results incomplete. Nevertheless, the results obtained are indicative and might lead to new guidelines and policy change, which will improve EMS activities in the future.

## Competing Interests

The authors have declared that no competing interests exist.

## Correspondence

Mahmoudreza Peyravi MD, Pre-hospital and Disaster Medicine Centre, Regionens HUS SE-40544, Gothenburg, Sweden, Sweden.

E-mail: mahmoudreza.peyravi@gu.se
