# Correction: Can Medical Decision-making at the Scene by EMS Staff Reduce the Number of Unnecessary Ambulance Transportations, but Still Be Safe?

**DOI:** 10.1371/currents.dis.ca2b7f102888bf394a9318c587b62043

**Published:** 2015-07-09

**Authors:** PLOS Currents

## Correction

The affiliation for the first author is incorrect. The complete, correct affiliation is: Prehospital and Disaster Medicine Centre, Department of Surgery, Sahlgrenska Academy, Gothenburg University, Gothenburg, Sweden; Faculty of Shiraz University of Medical sciences, Shiraz, Iran.

## Reference

Peyravi M, Örtenwall P, Khorram-Manesh A. Can Medical Decision-making at the Scene by EMS Staff Reduce the Number of Unnecessary Ambulance Transportations, but Still Be Safe?. PLOS Currents Disasters. 2015 Jun 30 . Edition 1. doi: 10.1371/currents.dis.f426e7108516af698c8debf18810aa0a.

View Article

